# Prevalence of and factors associated with high atherogenic index among adults in Nairobi urban informal settlements: The AWI-Gen study

**DOI:** 10.1371/journal.pgph.0000224

**Published:** 2022-07-15

**Authors:** David Wambui, Shukri Mohamed, Gershim Asiki

**Affiliations:** 1 Department of Public Health, East Carolina University, Greenville, North Carolina, United States of America; 2 Health and Systems for Health Unit, African Population and Health Research Center (APHRC), Nairobi, Kenya; 3 Department of Women’s and Children’s Health, Karolinska Institute, Stockholm, Sweden; Universiti Malaya, MALAYSIA

## Abstract

Dyslipidemia is an important cardiovascular disease predictor. Atherogenic index of plasma (AIP), a ratio of triglycerides (TG) to high density lipoprotein (HDL) cholesterol has been deemed to be more informative as a cardiovascular disease predictor compared to using any single predictor. The aim of this study was to explore the factors associated with elevated atherogenic index among people living in low-income urban areas of Nairobi, Kenya. Data used in this study were obtained from a cross-sectional population-based study with 2,003 participants conducted in Nairobi as part of the Africa Wits-INDEPTH Partnership for Genomic Research, AWI-Gen). Sociodemographic, behavioral, and clinical characteristics were collected from the participants. AIP was derived from the log of TG/HDL cholesterol and categorized into low risk (AIP<0.1), intermediate risk (AIP = 0.1–0.24) and high risk (AIP >0.24). Fifty-four percent (54%) of the study participants were women and the mean age of participants enrolled in this study was 48.8 years. Twenty-nine percent (29%) of study participants had high or medium atherogenic risk. Men, HIV patients, individuals with self-reported uncontrolled diabetes and obese individuals were at higher atherogenic risk. We have identified modifiable risk factors which can be addressed to reduce dyslipidemia in this population. Longitudinal studies may help to precisely determine how these factors relate with cardiovascular diseases.

## Introduction

Atherosclerotic cardiovascular diseases including ischemic heart disease, stroke and peripheral arterial disease present a significant contribution to global mortality [1]. In 2019, cardiovascular diseases accounted for 18.6 million deaths, a 17.1% increase since 2010 [2]. Cardiovascular disease has for the last 15 years consistently remained the leading cause of death globally [3]. The burden is projected to increase over the next couple of years [4–6]. By 2030 there will be 23.6 million deaths from heart disease and stroke [4]. Atherosclerotic cardiovascular diseases risk factors range from environmental, behavioral and clinical [7] causes including dyslipidemia, physical inactivity, obesity and Type II diabetes [8]. Understanding the distribution of these risk factors is critical in designing prevention strategies for atherosclerotic cardiovascular diseases. Individual lipid profiles as single measures have been used widely by clinicians and researchers as risk predictors for atherosclerotic cardiovascular diseases [8,9]. However, AIP has been shown to be more informative compared to single lipid profiles and hence a better risk predictor [7,8,10]. AIP has also been shown to reflect the distribution of lipid particle sizes and significantly correlates with presence of other atherosclerosis risk factors [11–13]. AIP takes into account the balance between harmful and protective lipids and has been described as a better risk predictor [10,14]. AIP is derived from a logarithmic transformation of the ratio between TG and HDL [15,16]. High values of AIP are associated with increased atherosclerotic cardiovascular risk. Over the recent past, atherogenic index, a ratio of triglycerides and HDL cholesterol has become an important predictor of cardiovascular diseases [17]. This is because unlike single lipid profile predictors, atherogenic index is more informative and is therefore regarded as a powerful surrogate indicator for cardiovascular disease risk [17,18]. A number of studies have shown correlations between high atherogenic index and cardiovascular diseases [7,10,15,19]. For better characterization of atherosclerotic cardiovascular risk using AIP, threshold values have been defined such that AIP of less than 0.1 regarded as low risk, 0.1 to 0.24 as intermediate risk and AIP above 0.24 regarded as high risk [10].

As a measure of atherosclerotic cardiovascular risk, it is important to understand factors that contribute to an increase in AIP. Over the past two centuries, epidemiological transition driven by industrialization, urbanization, economic growth, and lifestyle changes has resulted to the double burden of disease among sub-Saharan African countries with an upward trend in noncommunicable diseases. These changes have resulted to a cluster of environmental, behavioral and clinical risk factors for cardiovascular disease including hyperlipidemia, tobacco smoking, diabetes mellitus, hypertension, obesity, physical inactivity, alcohol consumption and poor dietary habits [20]. An interplay of these environmental and behavioral factors may explain the reported high prevalence of dyslipidemia (15–50%) among African adults [21]. It is projected that by 2030, cardiovascular diseases will be the leading cause of death overtaking infectious diseases [22].

These changes in environmental and behavioral factors among people in sub-Saharan Africa (SSA) could be a driver of increased atherosclerotic cardiovascular risk. A recent study reported a 50% increase in age-adjusted cardiovascular disease associated mortality in the region over the past three decades [23]. In 2017, SSA reported close to one million cardiovascular-related deaths with ischemic heart disease and stroke accounting for over 70% of these deaths [24]. In Kenya, a study conducted in the same population identified increased age and hypertension as significant drivers of CVD mortality in the population [25]. According to this study, addressing the identified risk factors would reduce the observed CVD mortality by up to 29%. This calls for better understanding of associated risk factors to better tailor preventative measures. The aim of this study was to explore the factors associated with elevated AIP among people living in low-income urban areas of Nairobi Kenya.

## Methods

### Study design and data collection

Data were drawn from a cross-sectional population-based study conducted in Nairobi as part of the Africa Wits-INDEPTH Partnership for Genomic Research, AWI-Gen. The AWI-Gen study was conducted to estimate physiological, environmental, and genetic risk factors for cardio-metabolic diseases among individuals aged between 40 and 60 years. This study focused on 2,003 participants who were recruited from two urban slums (Viwandani and Korogocho) in Nairobi, Kenya. Data collection occurred between 2014 and 2016. Participants were randomly selected until the target number was attained. Participants were randomly selected from an existing population sample [26]. Pregnant women, people with physical impairments that would prevent measurement of blood pressure and anthropometric indices and recent immigrants (those with less than 10 years of residency) were excluded from the study. A geographical sampling frame covering two peri-urban Nairobi slums, Korogocho and Viwandani were used to ensure an approximately equal selection from both slums as well as to ensure approximate equal number of male and female participants. Detailed participant enrollment procedures and sample size determination are included in the AWI-Gen study protocol [26,27].

### Ethics statement

The study was approved by the Human Ethics Committee of the University of Witwatersrand (Protocol Number: M121029) and African Medical Research and Education Foundation (AMREF)—Health Ethics and Scientific Review Committee in Kenya (P114-2014). All participants were provided with a paper consent form (in English or translated to Swahili) that described the study, ethical use of data, samples storage for future use, participant confidentiality and protection from harm. A copy is attached in supplementary materials.

### Measurements and definitions

A paper questionnaire (attached as supplementary materials) was administered by trained field interviewers to collect sociodemographic and behavioral characteristics including gender, age, ethnicity, occupation, education level, marital status, diet, physical activity, tobacco smoking and alcohol use. Sociodemographic and behavioral characteristics were all self-reported. Physical activity was self-reported using the Global Physical Activity Questionnaire (GPAQ) [28]. Dietary factors were measured using the WHO Steps Instrument [29] to assess the consumption of fruits and vegetables. Tobacco and alcohol use were self-reported including all forms of tobacco use (chewing, snuffing, smoking) or any form of alcohol (locally brewed or purchased) consumed. Weight and height were measured using digital Physician Large Dial 200kg capacity scales (Kendon Medical) and a stadiometer ((Holtain, Crymych, Wales) respectively. A soft measuring tape was used to measure waist and hip circumferences. These measurements were used to determine body mass index (BMI) and waist/hip ratio.

Blood pressure (BP) measurements were taken using a validated Omron™ M10-IT blood pressure machine with appropriately sized cuffs. A total of three BP measurements were taken with five minutes break between each measurement. Measurements were taken while participants were seated with their arm resting on armrest or a desk and arm facing up. We used the mean of the last two measurements. Hypertension was defined as either having a systolic blood pressure ≥ 140mmHg and/or a diastolic blood pressure ≥90mmHg, and/or a self-report of previous diagnosis of hypertension by a healthcare provider, and/or currently taking hypertension medication.

Fasting blood glucose (FBG), cholesterol levels (TG, HDL) were measured using a Randox Plus clinical chemistry analyser (UK) using colorimetric assays [26]. Diabetes was defined as a FBG ≥7 mmol/L or a random glucose of ≥11 mmol/L or a self-report of previous diagnosis of diabetes by a healthcare provider, or currently on treatment for diabetes. Tuberculosis infection was defined as a self-reported case of newly diagnosed tuberculosis i.e., in the last 12 months. HIV status was defined as a laboratory confirmed HIV positive test or self-reported to having ever tested HIV positive.

A LOGIQ e ultrasound system (USS) (GE Healthcare, CT, USA) with a 2–5 MHz 3C-RS curved array transducer were used to determine carotid intima-media thickness (CIMT), visceral adipose tissue and subcutaneous adipose tissue thicknesses. To measure CIMT, two sternocleidomastoid muscles were used as landmarks and the area was scanned to find the common carotid artery. CIMT was measured for the posterior of both left and right common carotid artery. To calculate the CIMT, the cursor was placed at two points of the posterior wall of about 10mm segment of the artery. The starting point was 1 cm from the bulb of the common carotid artery. CIMT measurement was automatically captured by the instrument as the distance of the intima-media interface [26]. Detailed explanation of biomarkers and other measurement is explained elsewhere by Ali et al. 2018 [26]. Atherogenic index was derived from the log of TG/HDL cholesterol and categorized into low risk (AIP<0.1), intermediate risk (AIP = 0.1–0.24) and high risk (AIP >0.24) [13].

### Statistical analysis

Descriptive statistics included means and standard deviation (SD) for normally distributed continuous variables and median and interquartile range (IQR) for continuous variables that were not normally distributed. Categorical variables were described using proportions stratified by gender. For continuous variables, a student’s t-test was conducted to determine differences between males and females while a chi-square test was performed for categorical variables. We conducted a univariate ordered logistic regression to determine socio-demographic, behavioral, biological, and clinical factors associated with AIP. From the univariate analysis, factors with a p-value of ≤ 0.1 were selected for the hierarchical stepwise approach ordered multivariate regression model [30]. The first model included only socio-demographic characteristics. From this model, only factors with p value <0.1 were included in the second model in addition to behavioral factors selected from the univariate models. The third model included all significant factors from the first and second models in addition to biological and clinical factors. The final model included age even though it was not statistically significant because age is a well-known risk factor for atherosclerosis. Multicollinearity of covariates included in the final model was conducted using the variable inflation factor (VIF) test. None of the covariates had a VIF >10 and therefore it was concluded that there was no multicollinearity in the model. Bayesian Information criterion (BIC) was used to determine changes in the overall fit of the models. Statistical analysis was performed using Stata 15 (Stata Corp, College Station, TX).

## Results

### Characteristics of study participants

As shown in (Table 1), the study enrolled 2,003 participants:1,081 (54%) women. The mean age of participants enrolled in this study was 48.8 years with a standard deviation of 0.13. The ethnic distribution showed that, 36% were Kikuyu, 20% Kamba, 16% Luhya, 19% were Luo and other ethnicities accounted for 9% of the study participants. In terms of behavioral characteristics, 29% reported tobacco use while 48% reported alcohol use. Most (64%) people reported no intake of sugary drinks. Although 93% of participants reported moderate-vigorous physical activity, 55% also reported that their work involves sedentary activities such as sitting or standing still. In terms of clinical characteristics, 7% of the participants had diabetes mellitus. 12% of participants were HIV positive, 12% had TB, 27% had hypertension and 18% had high total cholesterol. About 26% and 20% of the participants were overweight or obese respectively by BMI estimates while, 54% of participants had abnormal waist hip ratio (>0.95 cm for men and >0.80 cm for women) (87% among women and 16% among men). Subcutaneous fat and visceral fat were not normally distributed, and they had a median of 1cm (IQR 1-2cm) and 5cm (IQR 4cm-6cm) respectively. The overall mean right CIMT was 0.58 mm (SD, 0.12) while the overall mean left CIMT was 0.60 mm (SD, 0.12).

**Table 1:** Summary of characteristics included.

Sociodemographic characteristics	Behavioral characteristics	Clinical/Biological characteristics
Age	Tobacco use	Diabetes status
Ethnicity	Alcohol use	TB status
Marital status	Fruit/vegetable consumption	HIV status
Education	Vendor meals consumption	Hypertension status
Occupation	Sugary drinks consumption	Low density lipoprotein (LDL)
Wealth quintile	Sedentary work	BMI
	Physical activity	Subcutaneous fat
		Visceral fat CIMT
		Waist hip ratio

### Prevalence of atherogenic index

**Fig 1:**
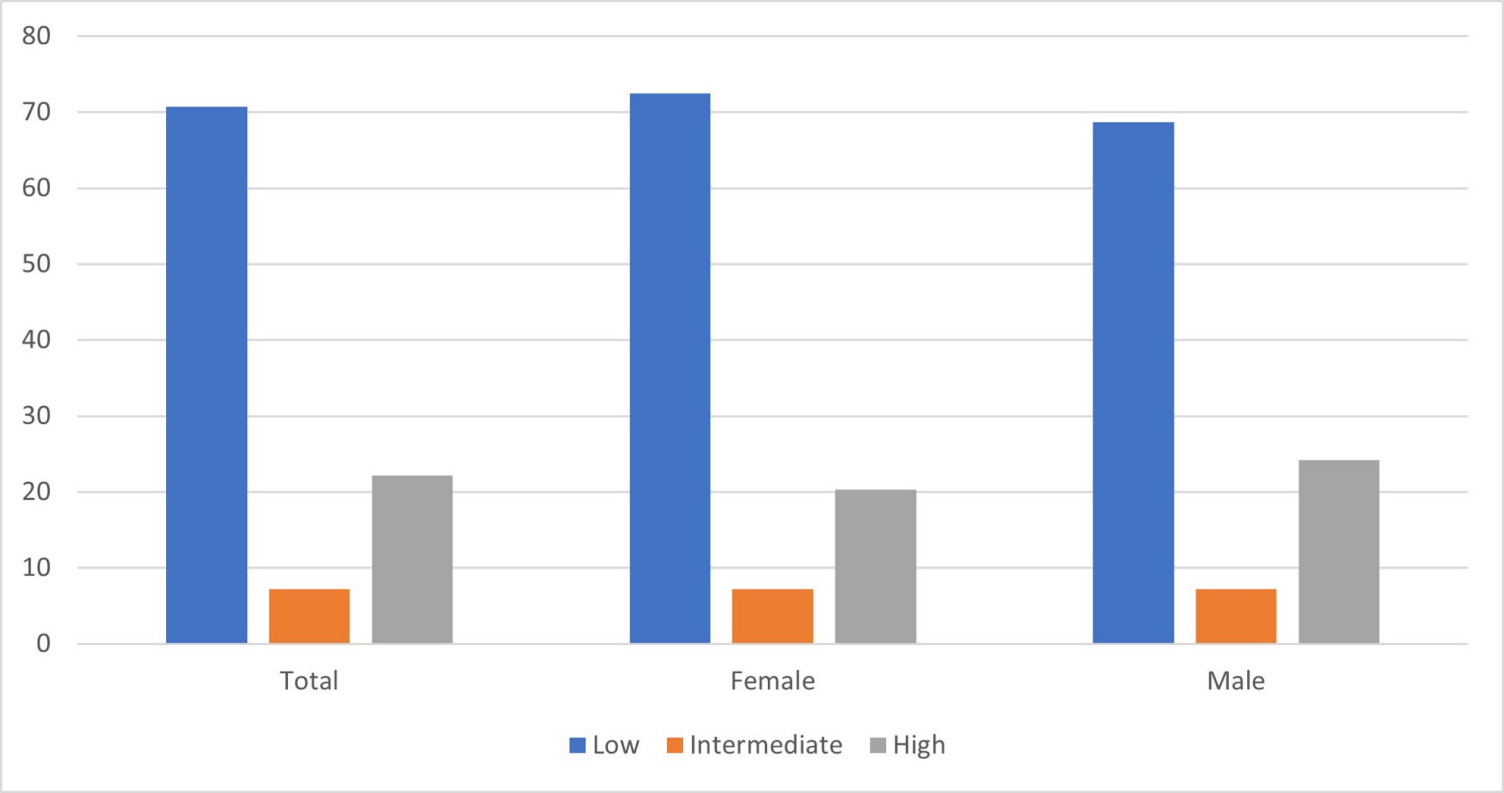
Atherogenic index among participants.

Most people (71%) had low atherogenic index. There were 7% (144) and 22% (443) of participants who had intermediate or high atherogenic index respectively (Fig 1). Men had a slightly higher proportion of high atherogenic index (24%) compared to women (20%). In terms of atherogenic risk distribution by age, those who were between 45 and 54 years accounted for 51% of individuals with high atherogenic index.

### Factors associated with atherogenic index

Univariate analysis showed that sex, age, ethnicity, occupation, socioeconomic status, sedentary lifestyle, diabetes, total cholesterol, LDL, BMI, subcutaneous fat, visceral fat, and left CIMT were associated with AIP (Table 2). Men were 1.21 times more likely to have higher atherogenic indices compared to women. Participants aged 55 years or older were 1.39 times more likely to have higher atherogenic index compared to those who were younger than 45 years. The Luhya or Luo ethnicities were 0.65 and 0.47 times less likely to have higher atherogenic index respectively compared to those from Kamba ethnic group.

**Table 2:** Sociodemographic, behavioral and clinical/biological characteristic of subjects.

Characteristic		Total	Female	Male	p-value
		N = 2,003	N = 1,081	N = 922	
Age (Years)	<45	564 (28%)	309 (29%)	255 (28%)	0.036
	45–54	1,043 (52%)	581 (54%)	462 (50%)	
	55 +	396 (20%)	191 (18%)	205 (22%)	
Ethnicity	Kamba	393 (20%)	196 (18%)	197 (21%)	<0.001
	Kikuyu	725 (36%)	480 (44%)	245 (27%)	
	Luhya	322 (16%)	143 (13%)	179 (19%)	
	Luo	374 (19%)	159 (15%)	215 (23%)	
	Other	189 (9%)	103 (10%)	86 (9%)	
Marital Status	Married/Cohabitating	1,332 (67%)	492 (46%)	840 (91%)	<0.001
	Never Married/ Divorced/Separated/ Widowed	670 (33%)	588 (54%)	82 (9%)	
Education	No Education	154 (8%)	118 (11%)	36 (4%)	<0.001
	Primary Level	1,151 (57%)	682 (63%)	469 (51%)	
	Secondary +	698 (35%)	281 (26%)	417 (45%)	
Occupation	Self-employed	946 (47%)	632 (58%)	314 (34%)	<0.001
	Formal (Full/Part-time)	312 (16%)	69 (6%)	243 (26%)	
	Informal	623 (31%)	287 (27%)	336 (37%)	
	Unemployed	119 (6%)	93 (9%)	26 (3%)	
Tobacco use	No	1,430 (71%)	998 (92%)	432 (47%)	<0.001
	Yes	573 (29%)	83 (8%)	490 (53%)	
Alcohol use	No	1,042 (52%)	778 (72%)	264 (29%)	<0.001
	Yes	960 (48%)	303 (28%)	657 (71%)	
Wealth quintile	First quintile	241 (12%)	145 (13%)	96 (10%)	<0.001
	Second quintile	451 (23%)	275 (25%)	176 (19%)	
	Third quintile	464 (23%)	245 (23%)	219 (24%)	
	Fourth quintile	405 (20%)	226 (21%)	179 (19%)	
	Fifth quintile	442 (22%)	190 (18%)	252 (27%)	
Fruit or Vegetable consumption	<5 servings	739 (37%)	372 (34%)	367 (40%)	0.013
	5 + servings	1,264 (63%)	709 (66%)	555 (60%)	
Vendor meals consumption		1 (0–3)	1 (0–2)	2 (0–4)	<0.001
Sugary drinks (No. of cups/bottles/cans)	0	1276 (64%)	749 (69%)	527 (57%)	<0.001
	1	519 (26%)	245 (23%)	274 (30%)	
	>1	208 (10%)	87 (8%)	121 (13%)	
Work Sedentary	No	901 (45%)	466 (43%)	435 (47%)	0.068
	Yes	1,102 (55%)	615 (57%)	487 (53%)	
Moderate—Vigorous PA	Inactive	141 (7%)	103 (10%)	38 (4%)	<0.001
	Active	1,862 (93%)	978 (90%)	884 (96%)	
Diabetic	No	1,843 (93%)	972 (91%)	871 (95%)	<0.001
	Yes	143 (7%)	99 (9%)	44 (5%)	
TB status	Negative	1,784 (89%)	964 (89%)	820 (89%)	0.86
	Positive	219 (11%)	117 (11%)	102 (11%)	
HIV status	Negative	1,536 (77%)	821 (76%)	715 (78%)	<0.001
	Positive	243 (12%)	175 (16%)	68 (7%)	
	Not known	224 (11%)	85 (8%)	139 (15%)	
Hypertensive	No	1,470 (73%)	757 (70%)	713 (77%)	<0.001
	Yes	533 (27%)	324 (30%)	209 (23%)	
Cholesterol level	Desirable	1,640 (82%)	867 (80%)	773 (84%)	0.035
	High	363 (18%)	214 (20%)	149 (16%)	
LDL		3.03 (1.14)	3.03 (1.13)	3.03 (1.16)	0.89
HDL		1.26 (0.47)	1.25 (0.44)	1.27 (0.50)	0.25
BMI	Underweight	149 (7%)	41 (4%)	108 (12%)	<0.001
	Normal	943 (47%)	360 (33%)	583 (63%)	
	Overweight	513 (26%)	333 (31%)	180 (20%)	
	Obese	398 (20%)	347 (32%)	51 (6%)	
Subcutaneous fat		1.57 (0.76)	1.94 (0.73)	1.14 (0.55)	<0.001
Triglycerides		1.09 (0.70)	1.07 (0.62)	1.12 (0.78)	0.04
Visceral fat		4.98 (3.72–5.99)	4.78 (3.59–5.81)	5.22 (3.96–6.28)	<0.001
Mean CIMT (Right)		0.58 (12)	0.58 (0.12)	0.57 (0.12)	0.079
Mean CIMT (Left)		0.60 (0.12)	0.61 (0.12)	0.60 (0.12)	0.028
Waist hip ratio	Normal	913 (46%)	140 (13%)	773 (84%)	<0.001
	Abnormal	1,090 (54%)	941 (87%)	149 (16%)	

** Data are presented as mean (SD) or median (IQR) for continuous measures, and n (%) for categorical measures.

Higher Socioeconomic status (SES) as indicated by wealth quintiles was associated with higher odds of high atherogenic indices. Similarly, increased use of sugary drink was associated with higher odds of high atherogenic indices with those who reported drinking one sugary drink having 0.08 higher odds and those who reported drinking more than one sugary drink having 0.37 higher odds of high atherogenic index compared to those who reported not drinking sugary drinks. High levels of subcutaneous and visceral fat were associated with higher odds of high atherogenic index. Those with abnormal waist-hip-ratio were 1.64 more likely to have high atherogenic risk.

From the multivariate models, model 3 was selected as it had the lowest BIC statistic and therefore provided the best fit for the data. The model indicated that male participants were about three times more likely to have higher atherogenic index compared to females. Luhya and Luo ethnicities were 34% and 54% less likely to have higher atherogenic index compared to Kamba ethnicity. Participants with diabetes had higher odds (2.23) of having a high atherogenic index compared to those without diabetes. Participants with HIV were 1.83 more likely to have a higher atherogenic index compared to those who were HIV negative. Individuals with high level of LDL were 22% more likely to have higher atherogenic index compared to those with desirable levels. Increased subcutaneous and visceral fat was associated with a high atherogenic index of 1.36 and 1.18 respectively. Lastly participants with abnormal waist hip ratio were 2.37 times more likely to be associated with a higher atherogenic index (Table 3). As shown in the hierarchical regression model (Table 4), sociodemographic characteristics only accounted for 2.4% of observed variance (R^2^ = 0.0235). Behavioral factors seemed to not influence the observed variance of atherogenic risk and clinical factors contributed additional 4.9% of the observed variance (ΔR^2^ = 0.0493).

**Table 3:** Univariate ordered logistic regression models of factors associated with higher atherogenic index.

Characteristic		OR (95% CI)	p-value	LR statistic p-value
**Demographic and socioeconomic factors**				
Sex	Female	1	0.05	0.05
	Male	1.21 (1.00–1.47)		
Age (Years)	<45	1		0.05
	45–54	1.08 (0.86–1.35)	0.52	
	55 +	1.39 (1.06–1.83)	0.02	
Ethnicity	Kamba	1		<0.001
	Kikuyu	1.00 (0.77–1.29)	0.98	
	Luhya	0.65 (0.47–0.90)	0.01	
	Luo	0.47 (0.34–0.65)	<0.001	
	Other	0.91 (0.63–1.32)	0.61	
Marital Status	Married/Cohabitating	1		0.56
	Never Married/ Divorced/Separated /Widowed	0.94 (0.77–1.15)	0.56	
Education	No Education	1		0.81
	Primary Level	1.07 (0.73–1.55)	0.73	
	Secondary +	1.12 (0.76–1.65)	0.56	
Occupation	Self-employed	1		0.003
	Formal (Full/Part-time)	0.81 (0.61–1.06)	0.13	
	Informal	0.66 (0.52–0.82)	<0.001	
	Unemployed	0.79 (0.52–1.21)	0.28	
Wealth quintile	First quintile	1		0.002
	Second quintile	1.54 (1.07–2.22)	0.02	
	Third quintile	1.66 (1.15–2.39)	0.01	
	Fourth quintile	1.46 (1.00–2.12)	0.05	
	Fifth quintile	2.06 (1.43–2.95)	<0.001	
**Behavioral Factors**				
Tobacco use	No	1		0.23
	Yes	1.14 (0.92–1.40)	0.23	
Alcohol use	No	1		0.13
	Yes	1.16 (0.96–1.40)	0.13	
Fruit or Vegetable consumption	<5 servings	1		0.27
	5 + servings	1.12 (0.92–1.36)	0.27	
Vendor meals consumption		0.99 (0.95–1.04)	0.76	0.76
Sugary drinks (No. of cups/bottles/cans)	0	1		0.13
	1	1.08 (0.86–1.35)	0.5	
	>1	1.37 (1.01–1.85)	0.04	
Work Sedentary	No	1		0.05
	Yes	1.21 (1.00–1.47)	0.05	
Moderate—Vigorous PA	Inactive	1		0.42
	Active	0.86 (0.60–1.24)	0.42	
**Clinical/Biological factors**				
Diabetic	No	1		<0.001
	Yes	2.82 (2.02–3.93)	<0.001	
TB status	Negative	1		0.26
	Positive	0.84 (0.61–1.15)	0.27	
HIV status	Negative	1		0.11
	Positive	1.33 (1.00–1.76)	0.05	
	Not known	0.91 (0.67–1.25)	0.56	
Hypertensive	No	1		0.07
	Yes	1.22 (0.99–1.51)	0.07	
LDL		1.30 (1.17–1.45)	<0.001	<0.001
BMI	Underweight	1		<0.001
	Normal	1.53 (0.98–2.39)	0.06	
	Overweight	2.66 (1.68–4.20)	<0.001	
	Obese	2.82 (1.77–4.50)	<0.001	
Subcutaneous fat		1.49 (1.32–1.69)	<0.001	<0.001
Visceral fat		1.29 (1.22–1.37)	<0.001	<0.001
Mean CIMT (Right)		1.41 (0.65–3.08)		0.39
Mean CIMT (Left)		2.14 (1.01–4.57)	0.05	0.05
Waist hip ratio	Normal	1		<0.001
	Abnormal	1.64 (1.35–1.99)	<0.001	

**Table 4:** Multivariate ordered regression models of factors associated with higher atherogenic index.

		Model 1	Model 2	Model 3
Characteristic		OR (95% CI)	OR (95% CI)	OR (95% CI)
Sex	Female	1	1	1
	Male	1.40 (1.13–1.73) ***	1.40 (1.13–1.73) **	3.42 (2.45–4.81) ***
Age (Years)	<45	1	1	
	45–54	1.10 (0.88–1.39)	1.10 (0.88–1.39)	
	55 +	1.40 (1.06–1.87) **	1.39 (1.05–1.85) *	
Ethnicity	Kamba	1	1	1
	Kikuyu	1.02 (0.78–1.33)	1.02 (0.78–1.33)	0.94 (0.72–1.24)
	Luhya	0.65 (0.47–0.91) **	0.65 (0.47–0.91) **	0.66 (0.47–0.93) *
	Luo	0.45 (0.32–0.64) ***	0.45 (0.32–0.64) ***	0.46 (0.32–0.66) ***
	Other	0.90 (0.62–1.31)	0.90 (0.62–1.31)	0.99 (0.67–1.46)
Occupation	Self-employed	1	1	
	Formal (Full/ Part-time)	0.73 (0.54–0.99) *	0.74 (0.55–1.00)	
	Informal	0.71 (0.56–0.91) **	0.72 (0.56–0.91) **	
	Unemployed	0.83 (0.54–1.28)	0.84 (0.54–1.29)	
Wealth quintile	First quintile	1	1	
	Second quintile	1.56 (1.07–2.27) *	1.56 (1.07–2.27) *	
	Third quintile	1.56 (1.08–2.26) *	1.55 (1.07–2.25) *	
	Fourth quintile	1.33 (0.91–1.96)	1.33 (0.91–1.96)	
	Fifth quintile	1.73 (1.18–2.52) **	1.73 (1.18–2.53) **	
**Behavioral**				
Sedentary				
**Clinical**				
Diabetic	No			1
	Yes			2.23 (1.57–3.20) ***
HIV status	Negative			1
	Positive			1.83 (1.34–2.51) ***
	Not known			0.97 (0.67–1.42)
LDL	Desirable			1
	High			1.22 (1.08–1.37) **
Subcutaneous fat				1.36(1.12–1.59) **
Visceral fat				1.18 (1.12–1.26) ***
Waist hip ratio	Normal			1
	Abnormal			2.37 (1.72–3.27) ***
**R** ^**2**^		**0.0235**	**0.0235**	**0.0728**
**ΔR** ^**2**^			**0**	**0.0493**
**BIC**		**3120.011**	**3116.975**	**2882.938**

*Factors show in this table were significant factors only.

## Discussion

In this study we estimated the prevalence of high atherogenic index as a marker of cardiovascular disease risk among older adults living in low-income urban areas of Nairobi Kenya. We further examined factors associated with high atherogenic index. The results revealed that, up to one fifth (22%) of the study population had a high atherogenic index and 7% were classified to have intermediate atherogenic risk index. While there was only one study that used AIP in the region, our study was consistent with the population-based cohort study conducted in rural Uganda [25]. The study reported 25% of participants to have high atherogenic risk. Similar to our study, the study in Uganda found that males had the greater proportion of high atherogenic risk.

When we explored factors associated with high atherogenic indices we found men, and participants with diabetes, HIV infection, high LDL, high subcutaneous and visceral fat and high waist-hip ratio had higher odds of experiencing a high AIP. Ethnicity was observed to be significantly associated with lower atherogenic index among the Luhya and Luo ethnicities. While there are no studies that have explored ethnicity as a determinant for cardiovascular diseases in Kenya, differences in dietary habits may have contributed to these findings. While the Left CIMT was a statistically significant predictor of higher atherogenic index from the univariate analysis. Anatomical origin differences between the right and the left carotid arteries have been speculated to explain the observed differences. The origin differences could mean differences in flow intensities from the aortic arch [31]. Men were three times more likely to have higher atherogenic index compared to women. Similar findings were reported in a population study in rural Uganda where women were significantly less likely to have higher atherogenic index compared to men [25]. While there are studies that have found similar differences [25,32–34], the observed difference in this study could be due to behavioral characteristic such as tobacco and alcohol use that was observed to be more prevalent among males. These results highlight the need for targeted interventions for men in such settings.

Individuals with diabetes mellitus were twice as likely to have higher atherogenic index compared to those without this condition. These findings are consistent other study findings reporting correlations between type 2 diabetes and high atherogenic indices [35–37]. The proposed mechanism for the observed association is an increased oxidative stress and endothelia cell dysfunction [35]. Matsuzawa et al. (1995) observed that high concentrations of visceral fat could drive insulin resistance. Metabolism of visceral fat releases free fatty acids which gets into the portal circulation then into the liver where they can cause enhanced biosynthesis of lipids [38]. Insulin resistance on the other hand may induce hyperlipidemia and glucose intolerance which can lead to atherosclerosis [38].

HIV infection was found to increase the likelihood of high atherogenic index by 57 Although we did not have information on the use of Antiretroviral therapy (ART), the observed results could be due to use of ART as has been reported in other studies [39,40]. Although complex HIV regimens have caused significant repression of viral replication and have helped to increase patient survival times tremendously, the ART has been shown to induce morphological abnormalities such as redistribution of body fat (lipodystrophy) [41]. This is characterized by peripheral fat wasting, dorsocervical fat pad enlargement, increased visceral fat and breast hypertrophy among women [41]. Metabolic abnormalities such as hypertriglyceridemia and hypercholesterolemia have also been identified to result from ART use [42,43]. An alternative mechanism is the direct effect of HIV replication leading to altered lipid metabolism resulting from inflammation caused by HIV shown to be associated with higher atherogenic index [44].

Lastly, we found LDL, subcutaneous, visceral fat, and waist-hip ratio to be independently associated with higher atherogenic index. LDL cholesterol has been shown to cause fatty deposits in arteries which could result in blocking of blood and oxygen flow [45]. In this study, subcutaneous fat was shown to elevate the risk of cardiovascular disease outcome. Some studies have as well identified subcutaneous fat as a risk enhancing factor [46,47] while other studies have indicated that it could be protective against cardiovascular disease outcomes [48–50]. A study by Demerath et al. [51] may help explain these discrepancies. According to the study, different levels of visceral fat may result to a different association between subcutaneous fat and cardiovascular disease outcome. Visceral fat is metabolically active and secretes inflammatory markers such as adipocytokines [52,53], homeostasis and fibrinogen markers [54,55] and vascular endothelial growth factor [56]. These factors could play a role in cardiovascular risk manifestation among individuals. Waist-hip ratio is a marker for visceral obesity. A high waist-hip ratio is an indicator of deposition of fat around abdominal organs, blood vessels and other body organs such as the heart thus its association with a higher atherogenic index [57].

### Study strengths and limitations

The strength of this study is that it is the first study from an informal urban settlement in Kenya to estimate AIP and its determinants. Atherogenic index is not commonly used as an index in cardiovascular risk studies. There are not a lot of such studies that have been conducted to investigate the AIP profiles among African populations. In line with this, there was the lack of validated local cut-off points for AIP in the African population. This could lead to misclassification of individuals and therefore under or over estimations of their atherogenic indices. Lastly, this study was cross-sectional in nature thus casual associations cannot be made. Nonetheless, valuable information on the prevalence and factors associated with AIP was obtained which is useful in developing programs or interventions that will promote cardiovascular health.

## Conclusion and recommendation

This study is among the few studies from sub-Saharan Africa to highlight AIP prevalence and factors associated with high atherogenic risk in two urban informal settlements. The results indicate that men, individuals with diabetes, HIV patients and obese individuals are at higher risk of atherosclerosis. These risk factors can be addressed through targeted programs and interventions such as screening individuals during routine care for early detection of cardiovascular diseases, targeted education to increase awareness. Further research is also needed to explore other atherosclerosis risk factors this study did not assess. Longitudinal studies with the same study participants to assess cardiovascular disease outcomes related with AIP may improve the understanding of AIP risk profiles in the population. Similarly, studies with more precise measurements of behavioral characteristics such as alcohol intake and robust dietary assessment will improve the AIP risk profiling.

## Supporting information

S1 Appendix Information and consent form. (DOCX)

S2 Appendix Questionnaire. (PDF)
